# Gastrointestinal Basidiobolomycosis: A Case Series

**DOI:** 10.7759/cureus.55008

**Published:** 2024-02-26

**Authors:** Yaser Meeralam, Abdulrahman M Basfar, Adnan Alzanbagi, Abdulaziz Tashkandi, Wallaa Al Harthi, Firdos Saba, Mutaz Khairo, Saleh Alzhrani, Mohammed Shariff

**Affiliations:** 1 Gastroenterology, Digestive and Liver Center, King Abdullah Medical City, Makkah, SAU; 2 Pathology, King Abdullah Medical City, Makkah, SAU; 3 Radiology, King Abdullah Medical City, Makkah, SAU; 4 Surgery, Specialized Surgical Unit, King Abdullah Medical City, Makkah, SAU

**Keywords:** crohns disease, splendore–hoeppli phenomenon, voriconazole therapy, fungal infection, gastrointestinal basidiobolomycosis

## Abstract

Gastrointestinal basidiobolomycosis (GIB) is a rare fungal infection caused by Basidiobolus ranarum, a saprophytic fungus that belongs to the class of Basidiobolomycetes. It mainly infects immunocompetent individuals and is mainly found in arid tropical and subtropical regions, including Southwestern America, Saudi Arabia, Africa, and Asia. Not surprisingly, a great number of human infections have been reported from these warm, humid climate regions that are felicitous for the growth of this fungus, especially from the southern region of Saudi Arabia and Arizona in the United States of America. GIB is easily misdiagnosed as malignancy, inflammatory bowel disease, diverticulitis, lymphoma, and chronic intestinal infections due to its rarity. In this case series, we summarize the clinical features, imaging, histopathological features, and treatment of patients diagnosed with GIB in our institution.

## Introduction

Gastrointestinal basidiobolomycosis (GIB) is a rare fungal infection caused by Basidiobolus ranarum, an environmental saprophyte that is found worldwide, especially in arid regions [1-3]. This infection mainly affects the gastrointestinal tract and can present in various forms, such as colonic and hepatic masses [1,3,4]. GIB was considered an extremely rare disease until the late 1990s when only a few cases had been reported [1,3]. However, recent studies have shown the emergence of GIB in different parts of the world, including the United States and Saudi Arabia [1,4]. The increased number of reported cases may be partly due to the improved awareness and diagnosis of the disease, especially in endemic areas such as the southern province of the Kingdom of Saudi Arabia (KSA), where most cases have been detected. In this paper, we report five cases of GIB that were admitted to our tertiary care hospital with various gastrointestinal (GI) manifestations initially misdiagnosed as colonic malignancy, liver hemangioma, or inflammatory bowel disease.

## Case presentation

Case 1

A 23-year-old male with no known medical illnesses from a town in the southwest region of the KSA was referred to our hospital with a four-month history of intermittent chronic upper abdominal pain that was getting progressively worse. This was associated with bloody diarrhea and significant weight loss of about 15 kgs. There was no family history of cancer or surgery and the patient denied any recent travel.

**Figure 1:**
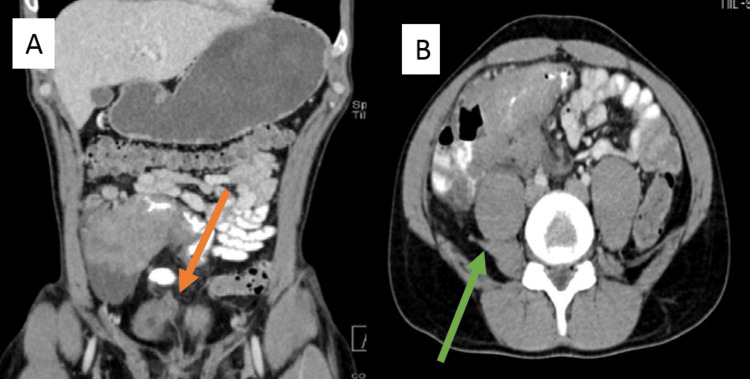
Contrast-enhanced CT scan (Case 1) Contrast-enhanced CT scan (A: coronal plane, B: axial plane) demonstrates marked irregular thickening of the terminal ileum with a mass-like appearance (green arrow) and ileocecal valve (orange arrow), associated with minimal ileocolic mesenteric inflammatory fat stranding. Terminal ileum luminal narrowing secondary to the mural thickening.

On examination, the patient had a right iliac fossa mass with an eosinophil percentage of 26.9% of the total white blood cell count (WBC) of 14 x10⁹/L and an erythrocyte sedimentation rate (ESR) of 19 mm/L. CT abdomen showed irregular mural thickening of the distal ileum and caecum along with enlarged mesenteric lymph nodes, raising suspicion of malignancy (Figures 1A-1B).

**Figure 2:**
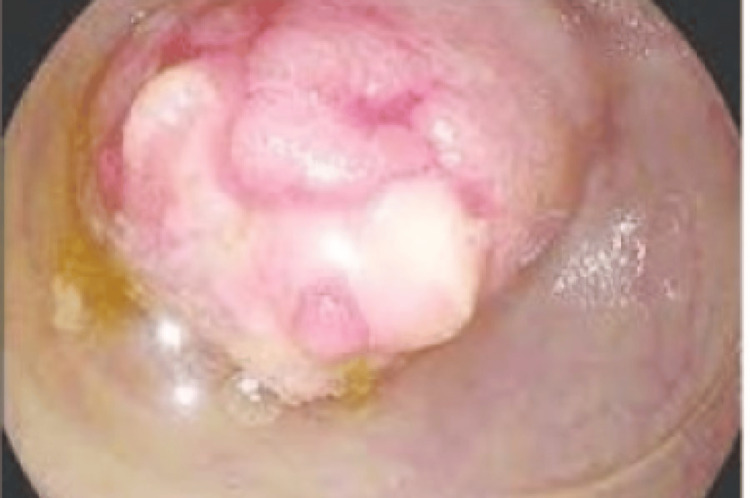
Colonoscopic image of a large cecal polypoidal lesion (Case 1)

At colonoscopy, a large polypoidal lesion was seen in the caecum (Figure 2) with granularity, patchy erythema, and ulceration of the terminal ileum.

**Figure 3:**
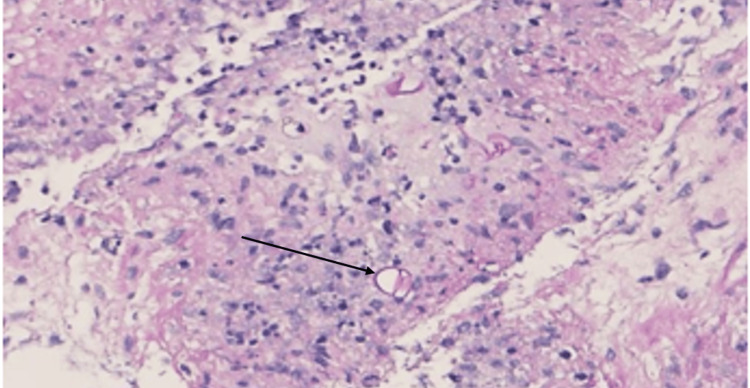
(Case 1 ) Rounded fungal zygospore Rounded fungal zygospore highlighted by periodic acid-Schiff stain; the arrow indicates the spore.

Histopathology of the polypoidal lesion biopsy showed chronic inflammation with eosinophilia. Stool examination and culture were negative for intestinal pathogens. The patient was treated symptomatically and discharged home with a follow-up in the outpatient clinic while awaiting further investigations. The culture of colonoscopy biopsy tissue was negative for tuberculosis and fungi. However, two months later the patient was readmitted with partial intestinal obstruction requiring right hemicolectomy. The histopathology of the resected specimen was described as showing polymorphous infiltration with eosinophils, and few plasma cells, in addition to many fungal hyphae surrounded intensely by eosinophilic granular radiating material with focal collection of epithelioid cells and foreign body type of giant cells in the adjacent soft tissue (Figure 3).

These morphological features were highly suggestive of Basidiobolomycosis. Hence, the patient was started on long-term oral itraconazole 200 mg with a planned duration of 18 months depending on the clinical progress. The patient was clinically well with no further pain or diarrhea on follow-up after four months.

Case 2

**Figure 4:**
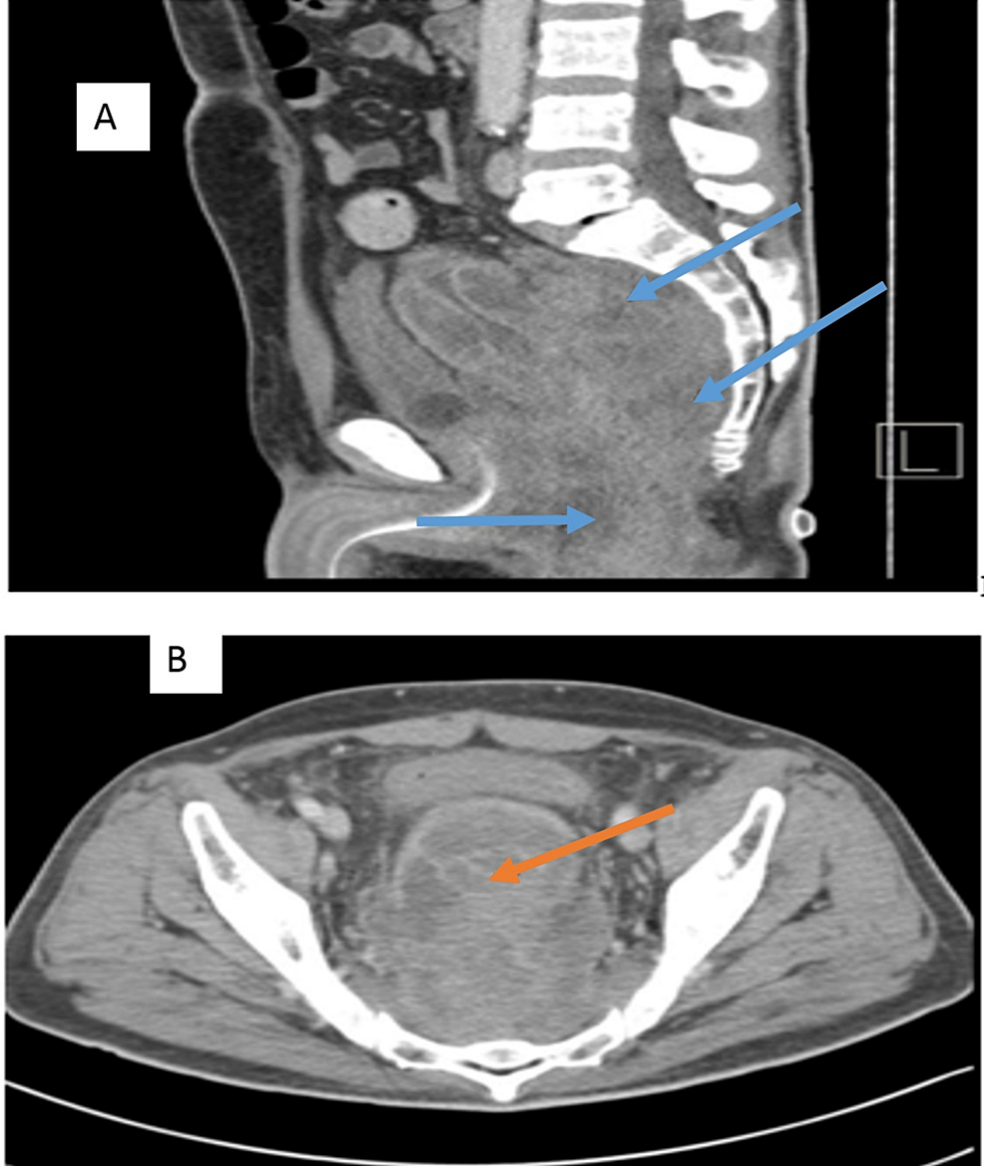
CT scan of the pelvis with intravenous contrast (Case 2) CT scan of the pelvis with intravenous contrast (A: sagittal plane, B: axial plane) demonstrates heterogeneously enhancing circumferential rectal mass (blue arrows) infiltrating the mesorectal fat and pre-sacral region (orange arrow), associated with pelvic fat stranding.

A 52-year-old male patient, a known case of diabetes mellitus type 2 residing in a southwestern town of KSA, was referred from a secondary hospital with symptoms of one-month duration of recurrent lower abdominal pain associated with bloody stool, tenesmus, and weight loss. A digital rectal exam revealed a nodular circumferential mass approximately three centimeters from the anal verge. Laboratory investigation showed an eosinophil percentage of 20.0% of a WBC of 17x 10⁹/L and an ESR of 90 mm/L. The stool examination and cultures were negative for intestinal pathogens. CT scan of the abdomen reported an extensive circumferential wall thickening/mass of the rectum, 13 cm in length with extramural soft tissue extension (Figures 4A-4B).

**Figure 5:**
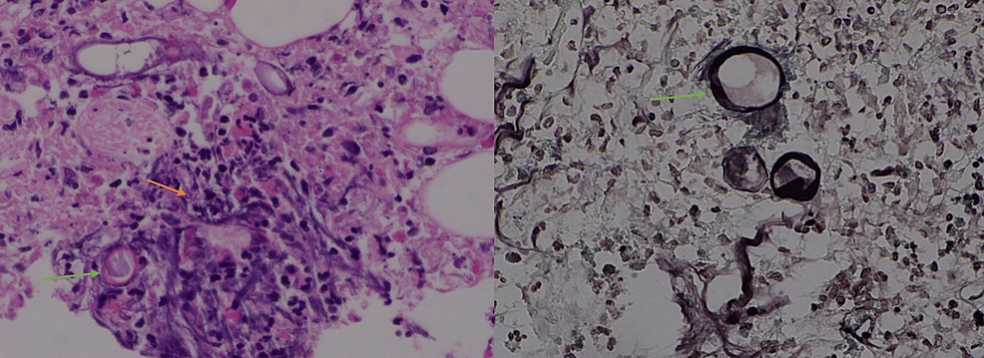
Histopathology showing septate fungal hyphae (Case 2) A: Histopathology magnification x40, hematoxylin, and eosin stain. Areas of inflammation with many broad, sparsely septate fungal hyphae along with few fungal rounded zygospores with thin outer walls can be seen (orange arrow - fungal hyphae; green arrow - spore, basidiobolomycosis); B: magnification of 100x, Grocott methenamine silver (GMS). The green arrow indicates the fungal spore.

An attempted colonoscopy was unsuccessful due to a very tight anorectal stricture impeding the endoscope. Multiple biopsies were taken from the stricture and the histopathology showed areas of inflammation composed of eosinophils and many broad, sparsely septate fungal hyphae. In addition, rounded zygospores with thin outer walls, foamy cytoplasm, and nucleus were noted (Figures 5A-5B). No features of malignancy were seen.

**Figure 6:**
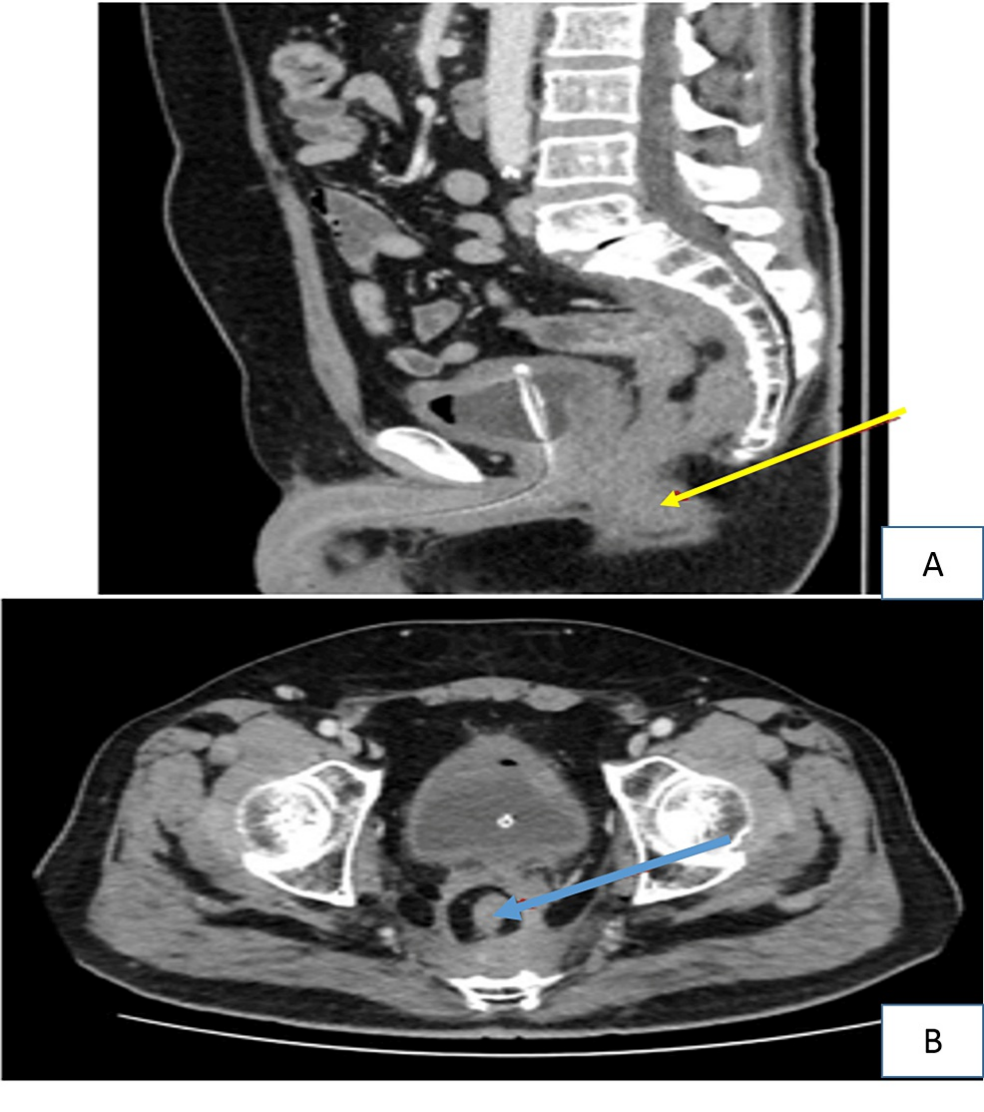
CT scan of the pelvis with intravenous contrast (Case 2) CT scan of the pelvis with intravenous contrast (A: sagittal plane; B: axial plane) after treatment showed significant regression of the circumferential rectal mass post-treatment (yellow arrow). Mild residual mesorectal fascia thickening (blue arrow) and pre-sacral infiltration can be seen.

The patient was diagnosed with GIB based on histopathologic findings representing the Splendore-Hoeppli phenomenon and was started on voriconazole and was requested to continue it long-term for a planned duration of 18 months depending on the clinical progress. Colonoscopy biopsy tissue sent for fungal culture was negative for any growth. During the outpatient follow-up six months later, the patient's symptoms had improved on voriconazole and a repeat CT scan of the pelvis showed a significant regression of the rectal mass (Figures 6A-6B).

Case 3

**Figure 7:**
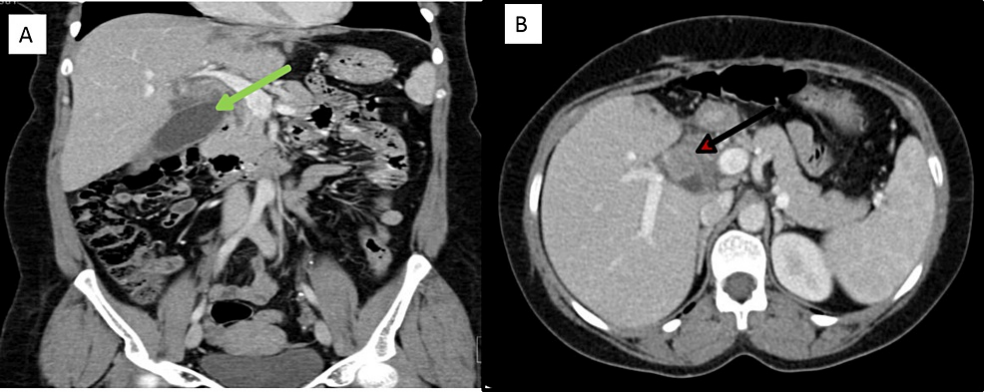
CT scan of the abdomen with IV contrast (Case 3) CT scan of the abdomen with IV contrast (A: coronal plane, B: axial plane) showing a 4 cm heterogeneously enhancing porta hepatis soft tissue mass involving the gallbladder neck and cystic duct (green arrows). There is diffuse CBD thickening and mural enhancement (black arrow). The left liver lobe was surgically removed.

A 41-year-old woman was referred to our hospital following a left hepatic lobe resection that was carried out for a mass lesion (details of which were not available), with a persistent porta hepatitis mass for further workup. The lady complained of intermittent epigastric pain for two months associated with vomiting. She denied any history of diarrhea, jaundice, or fever. On examination, she had a left upper quadrant tender mass. The patient’s laboratory investigations included an eosinophil percentage of 18.0% of a WBC of 13 x10⁹/L and ESR of 83 mm/L. CT scan of the abdomen showed a large left hepatic lobe mass with peripheral nodular enhancement measuring 9.7x7.7x6.7 cm suggestive of partially thrombosed hemangioma (Figures 7A-7B).

**Figure 8:**
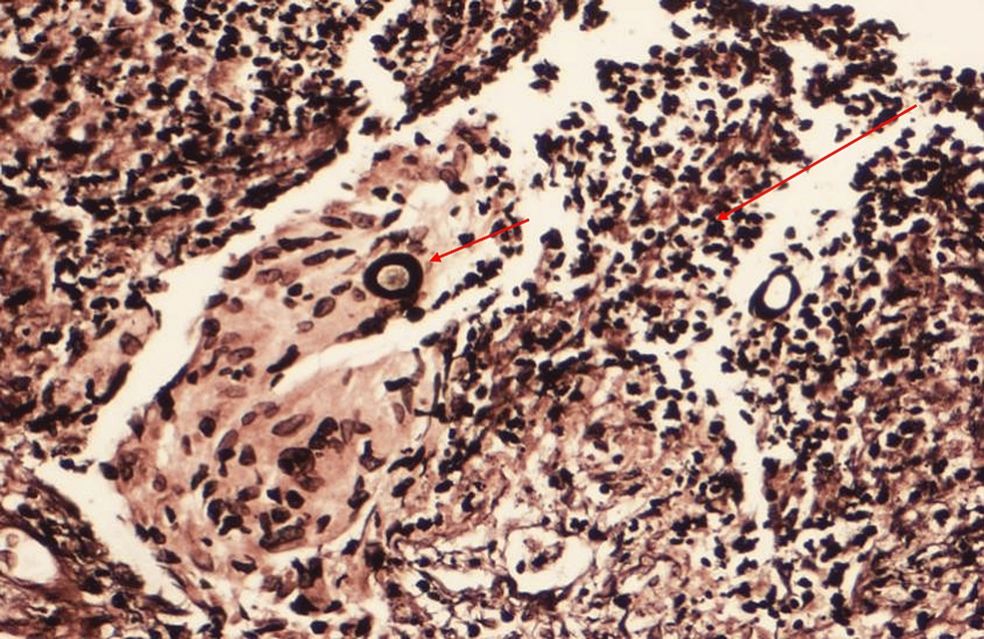
Histopathology (Case 3) GMS stain, magnified at 40x showing Basidiobolomycosis from the liver with fungi surrounded by acute inflammatory cells. The large arrow indicates the Splendore-Hoeppli phenomenon, and the red arrow shows spores of the fungus.

Ultrasound-guided biopsy from the liver lesion was obtained. The histopathology showed inflammatory cells, numerous eosinophils, and occasional multinucleated giant cells. Fungal hyphae were seen on periodic acid-Schiff stain and Grocott methenamine silver stain showing the typical Splendore-Hoeppli phenomenon, suggestive of Basidiobolomycosis (Figure 8).

**Figure 9:**
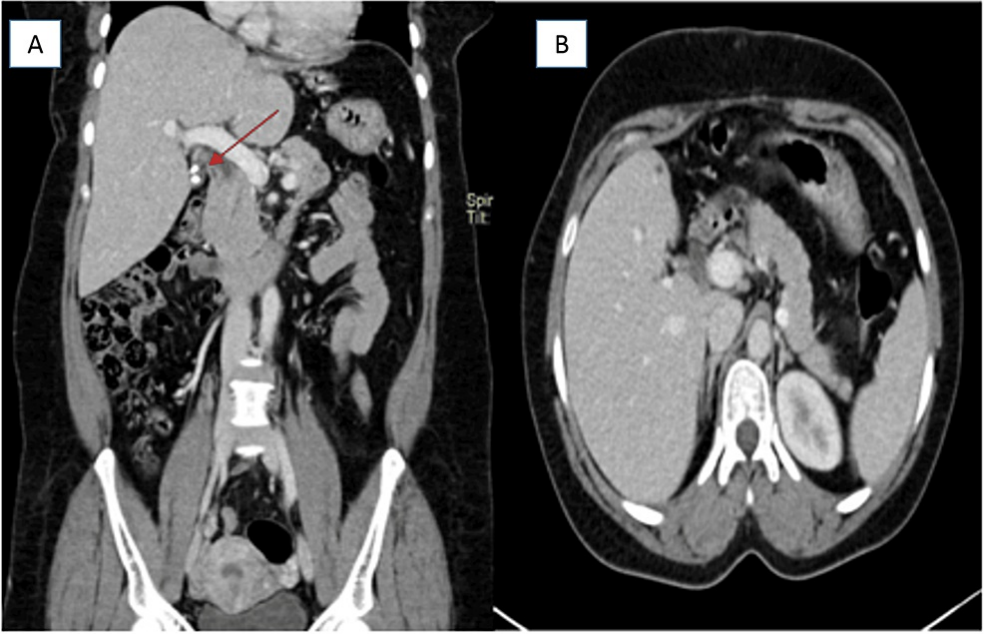
CT scan of the abdomen with IV contrast (Case 3) CT scan of the abdomen with IV contrast (A: coronal plane, B: axial plane). Post antifungal treatment and cholecystectomy show resolution of the porta hepatic mass and cholecystectomy surgical clips (red arrow).

The tissue sent for fungal culture was negative for any growth. She was started on itraconazole with a plan for long-term treatment depending on clinical progress. The patient was asymptomatic and a follow-up in the clinic and a repeat CT scan showed improvement in the size of the porta hepatis mass (Figure 9).

Case 4

**Figure 10:**
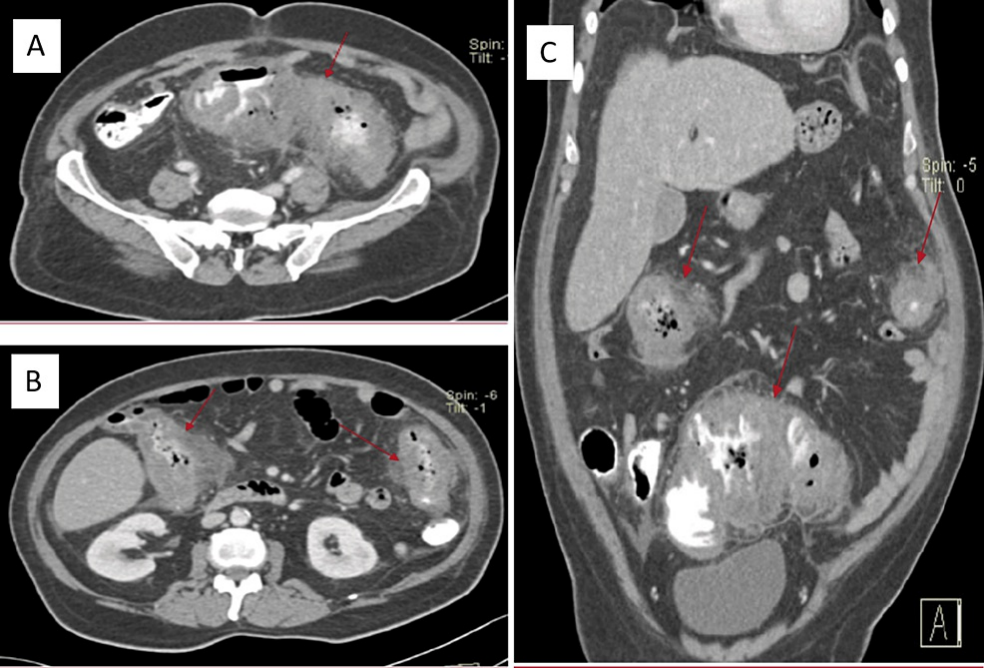
Abdominal contrast-enhanced CT scan (Case 4) Contrast-enhanced CT scan (A, B: axial plane, C: sagittal pane) demonstrates multifocal marked irregular segmental thickening involving the hepatic flexure, splenic flexure, and sigmoid colon, associated with pericolonic inflammatory fat stranding and luminal narrowing (red arrows). No bowel obstruction.

A 54-year-old male with a history of type 2 diabetes mellitus and chronic kidney disease was referred to our hospital with suspected colorectal neoplasia with partial large bowel obstruction. He had presented with a two-month history of lower abdomen pain, change in bowel habits, significant weight loss, generalized fatigue, and intermittent fever. There was no family history of cancer, no surgical history and the patient denied any recent travel. On physical examination, the patient had mild tenderness at the left iliac fossa without palpable mass or lymphadenopathy. His investigations revealed a WBC of 13 x10⁹/L, eosinophil percentage of 11.5 %, and ESR of 42mm/h. Abdominal CT scan showed multiple colonic masses involving the sigmoid and ascending colon with wall thickness and local extension surrounded by fat stranding (Figure 10).

**Figure 11:**
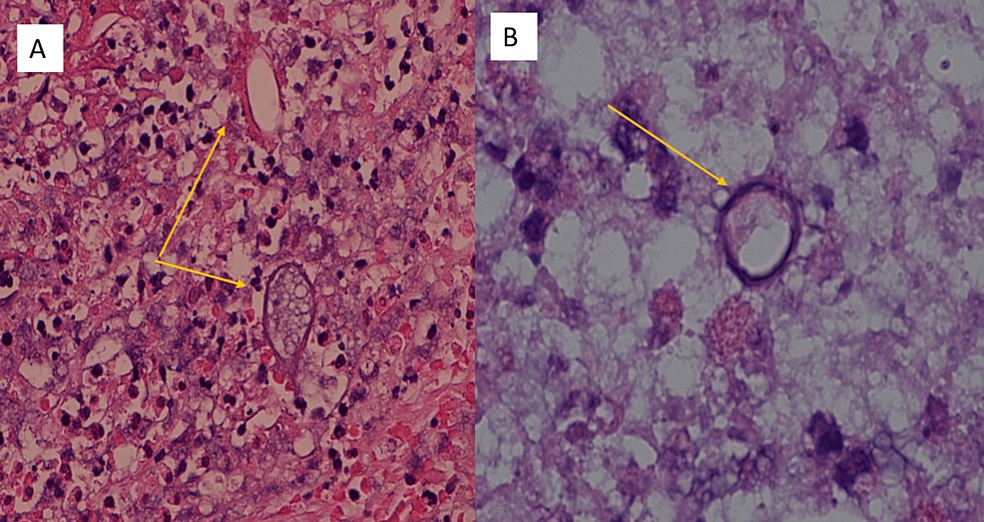
Histopathology for fungus (Case 4) A: H&E stain, X40, showing inflammatory nodular lesions with fungal colonies amidst inflammation and necrosis (Basidiobolomycosis indicated by orange arrows B: X100, H&E stain showing Basidiobolomycosis

A colonoscopy performed revealed large multiple polypoidal lesions extending distally from 16 cm to 34 cm, causing stricturing and impeding the endoscope at this point. Multiple biopsies of the lesion showed inflammation and granulation without a conclusive diagnosis. After a multidisciplinary meeting, a total colectomy with ileorectal anastomosis was performed. The histopathology using H&E stain showed polymorphous infiltration with numerous eosinophilic granular material and plasma cells with fungal spores surrounded by necrotic cells characteristic of the Splendore-Hoeppli phenomenon (Figures 11A-11B).

Multiple specimens from the colon sent for fungal culture were negative for any growth. Based on the histopathological features the patient was treated with oral voriconazole for the long term. The patient was asymptomatic and clinically well at a follow-up six months later.

Case 5

**Figure 12:**
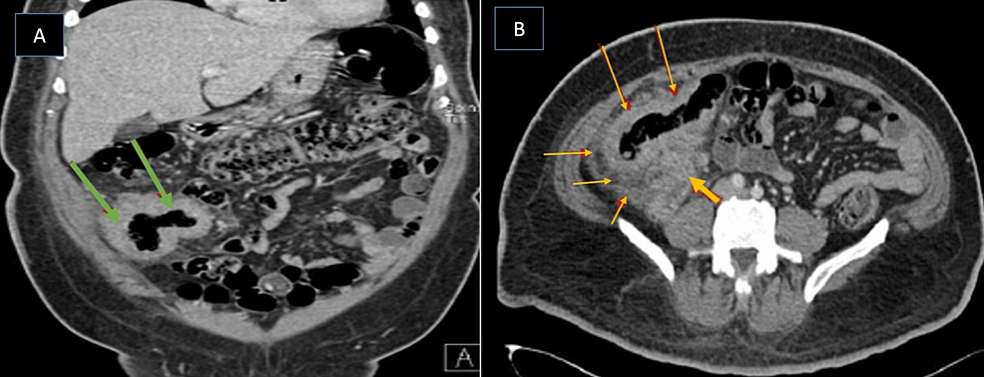
Enhanced CT scan of the abdomen and pelvis (Case 5) A and B: Enhanced CT scan of the abdomen and pelvis (A: sagittal plane, B: axial plane) shows marked irregular thickening of the terminal ileum and cecum (long arrows) with pericolonic inflammatory fat stranding (orange arrows) and subcentimeter lymph nodes. The appendix (green arrows) is diffusely thickened with heterogeneous enhancement as well.

A 58-year-old female patient from the southern region of KSA with diabetes mellitus type 2 was referred with non-bloody diarrhea of four weeks duration associated with diffuse abdominal pain. She denied any history of fever, loss of weight, loss of appetite, or night sweats. She had no family history of cancer and denied any recent travel. Abdominal examination revealed diffuse tenderness, with a palpable firm mass in the right lower quadrant of the abdomen. Laboratory tests showed an eosinophil percentage of 12.7% of the total WBC of 7.79 x 109 /L and an ESR of 52 mm/h. A CT scan of the abdomen showed segmental mural wall thickening affecting the transverse colon, the ascending colon, the cecum, and the terminal ileum with an inflammatory phlegmon at the right iliac fossa forming entero-enteric fistula (Figures 12A-12B).

**Figure 13:**
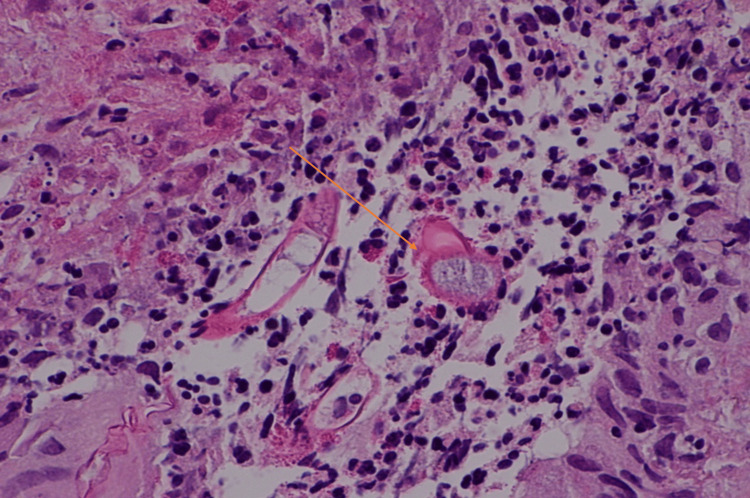
Histopathology fungal colony (Case 5) Magnification x40, periodic acid-Schiff stain. The active inflammation with fungal colony is indicated by the orange arrow. Broad, sparsely septate fungal hyphae are present amidst neutrophils (Splendore-Hoeppli phenomenon). A few epithelioid cells can be seen at the peripheries.

CT-guided biopsy of the phlegmon showed non-specific chronic granulomatous inflammation. The patient was started on voriconazole 200 mg on a presumptive diagnosis of GIB. Two weeks after starting the anti-fungal, at outpatient follow-up, the patient had no abdominal pain and her diarrhea had resolved. The patient was advised to continue anti-fungal treatment until further follow-up in six months. Two months later, the patient presented to the emergency department with severe abdominal pain with guarding and rigidity. An urgent CT scan of the abdomen revealed a cecal perforation. The patient underwent an exploratory laparotomy that confirmed a retroperitoneal cecal perforation and an extended right hemicolectomy with end ileostomy was performed. The histopathology of the resected specimen exhibited multiple granulomas composed of epithelioid cells with areas of necrosis and inflammation predominantly of eosinophils (Splendore-Hoeppli phenomenon). Scattered broad, sparsely septate fungal hyphae were present in the granulomas. In addition, fungal rounded zygospores with thin outer walls, foamy cytoplasm, and nucleus were noted (Figure 13).

The patient was advised to continue voriconazole orally for the long term and follow up at the outpatient department. After three months, she was asymptomatic with good appetite. 

## Discussion

Our case series describes the various forms of GIB presentation, requiring a battery of investigations to diagnose and the typical therapy of a combination of antiviral medication and surgery for management. Immunocompetent individuals are the usual victims of GIB [5]. The pathogenesis of gastrointestinal basidiobolomycosis (GIB) in adults remains unclear. However, recent taxonomic studies based on antigenic analysis, isoenzyme banding, and restriction enzyme analysis have indicated that all human pathogens belong to Basidiobolus ranarum [6]. The fungus is believed to enter the gastrointestinal tract through the ingestion of contaminated soil or food, leading to the development of infection in the submucosal and muscular layers of the bowel. This results in the formation of granulomatous lesions and inflammatory masses [7,8]. The vast majority of the published cases were of males and children, suggesting potential gender- and age-related susceptibility factors [1,3,9]. The median age of adults with GIB is reported to be 43 years, with a range of 37 to 59 years, with the most common comorbidities of diabetes mellitus and peptic ulcer disease [1,9]. Likewise, we could not identify how our patients contracted the infection and three out of five of our GIB patients were men with a mean age of 40 years.

The clinical presentation of GIB in adults can mimic infiltrative, infectious, or inflammatory processes, posing diagnostic challenges [1,3]. GIB manifests with a range of symptoms, but the most common ones include abdominal pain, palpable abdominal mass, weight loss, and fever. Patients often experience abdominal pain as localized or diffuse with mild to severe intensity. The abdominal mass usually is firm and non-tender, corresponding to the fungal granuloma in the GI tract. The fever is usually of low grade. Importantly, these symptoms are nonspecific, meaning they can overlap with various other conditions. All our cases presented with abdominal pain and two of them had palpable abdominal mass, although none had fever. Thus, the differential diagnosis for GIB in adults encompasses a range of conditions due to its nonspecific clinical presentation, leading to misdiagnosis and delay. The main differential diagnoses include inflammatory bowel diseases (IBD) such as Crohn's disease (CD), intestinal tuberculosis, and other granulomatous diseases [10,11]. Additionally, GIB should be considered in the differential diagnosis of mucormycosis, particularly in cases of colonic masses and eosinophilic intestinal inflammation [12,13]. The condition may also mimic malignancies; furthermore, the presence of eosinophilic granulomatous tissue reaction prompts consideration of entomophthoromycosis and other fungal infections [1,13,14]. Most of our patients were externally evaluated and were initially misdiagnosed. Three of the patients were suspected of colonic malignancy, one was presumed to have hepatic hemangioma and one patient was suspected as case of fistulizing CD.

The diagnosis of GIB presents significant challenges due to the lack of a definitive diagnostic modality. Laboratory findings that should raise the suspicion of GIB consist of leukocytosis, marked eosinophilia, and elevated ESR as observed in all our reported cases [9,15]. Serologic tests, such as enzyme-linked immunosorbent assay or immunodiffusion, have shown promise for the presumptive diagnosis of GIB, particularly when used with proper controls [9,16]. Radiological imaging modalities such as CT or MRI show characteristic findings of multiple masses with surrounding inflammatory components like soft tissue stranding and bowel wall thickening. Occasionally abscess formation in the affected area can be seen appearing as a localized collection of pus or fluid; however, these are not specific to GIB [9,17]. Colonoscopy aids in the diagnosis, but endoscopic features are typical of inflammatory processes, such as ulcerated polypoidal or nodular mucosa with necrosis and exudates [1,10,11,14]. Biopsies of the involved organ, colon, or liver often show features of nonspecific inflammation. Nevertheless, if the yield of the specimen is enough it may demonstrate the typical histopathological features, such as eosinophilic granulomatous inflammation with thin-walled, septate, haphazardly branched hyphae surrounded by eosinophilic, amorphous material, exemplifying the Splendore-Hoeppli phenomenon [1,6-8,14]. This crucial histopathological feature aids in the accurate diagnosis of this rare fungal infection. However, only 50% of cases display this phenomenon, highlighting the need for alternative diagnostic modalities [9].

Microbiology cultures are considered the gold standard for diagnosis, but they may only be achieved in a limited number of cases [5]. All the patients in the current series had CT and/or MRI scans that showed multiple masses with inflammatory processes. Endoscopic strictures and polypoidal growth were evident in three of the patients who had colonoscopies, with two showing nonspecific inflammation and only one of the patient’s biopsies suggesting typical features of GIB. Histopathology of the surgically resected specimen was required to confirm the diagnosis in the third and remaining two cases. Cultures in all five cases were negative for GIB.

Treatment options for GIB in adults typically involve a combination of surgical intervention and antifungal therapy. Surgery, often in the form of resection, has been a mainstay in the management of GIB, with the majority of patients having antifungal therapy in conjunction [1,5,12]. This approach aims to achieve complete removal of the infected tissue and reduce the fungal burden within the gastrointestinal tract. Antifungal therapy, particularly with azoles such as itraconazole and voriconazole, has shown promise in the treatment of GIB, with reports of marked improvement and successful outcomes [1,2,5,9]. However, the duration of antifungal therapy for GIB is not well-established. Duration of therapy ranging from three to 19 months has been reported to produce marked improvement [1,5,9]. However, a duration of at least six months has been recommended. It is important to note that the choice and duration of antifungal therapy for GIB may vary based on individual patient factors and the extent of the infection. However, GIB can be an aggressive disease with a high mortality rate despite treatment, particularly in cases with concomitant liver involvement [1,12]. This highlights the need for early and aggressive management to improve outcomes.

Furthermore, the emergence of resistance to certain antifungal agents, such as amphotericin B, itraconazole, and fluconazole, underscores the importance of susceptibility testing and the consideration of alternative antifungal agents [16]. As exemplified in our reported cases of GIB, all patients had combined antifungal and surgical resection. In one of these cases, an attempt at treating just with antifungal was not sufficient and the patient required a resection to achieve a better outcome. The prognosis of GIB in adults remains relatively unknown, with further studies needed to better define risk factors and optimize treatment strategies [9].

## Conclusions

In conclusion, the rarity of the disease and its nonspecific clinical presentation contribute to diagnostic delays, potentially impacting the prognosis. Additionally, the challenges associated with diagnosing and treating GIB emphasize the need for increased awareness and understanding of this emerging fungal infection.
